# Deciphering drug resistance in *Mycobacterium tuberculosis* using whole-genome sequencing: progress, promise, and challenges

**DOI:** 10.1186/s13073-019-0660-8

**Published:** 2019-07-25

**Authors:** Keira A. Cohen, Abigail L. Manson, Christopher A. Desjardins, Thomas Abeel, Ashlee M. Earl

**Affiliations:** 1 Division of Pulmonary and Critical Care Medicine, Johns Hopkins University School of Medicine, Baltimore, MA 21205 USA; 2 Broad Institute of Harvard and Massachusetts Institute of Technology, 415 Main Street, Cambridge, MA 02142 USA; 3 Delft Bioinformatics Lab, Delft University of Technology, 2628 XE Delft, The Netherlands

## Abstract

Tuberculosis (TB) is a global infectious threat that is intensified by an increasing incidence of highly drug-resistant disease. Whole-genome sequencing (WGS) studies of *Mycobacterium tuberculosis*, the causative agent of TB, have greatly increased our understanding of this pathogen. Since the first *M. tuberculosis* genome was published in 1998, WGS has provided a more complete account of the genomic features that cause resistance in populations of *M. tuberculosis*, has helped to fill gaps in our knowledge of how both classical and new antitubercular drugs work, and has identified specific mutations that allow *M. tuberculosis* to escape the effects of these drugs. WGS studies have also revealed how resistance evolves both within an individual patient and within patient populations, including the important roles of de novo acquisition of resistance and clonal spread. These findings have informed decisions about which drug-resistance mutations should be included on extended diagnostic panels. From its origins as a basic science technique, WGS of *M. tuberculosis* is becoming part of the modern clinical microbiology laboratory, promising rapid and improved detection of drug resistance, and detailed and real-time epidemiology of TB outbreaks. We review the successes and highlight the challenges that remain in applying WGS to improve the control of drug-resistant TB through monitoring its evolution and spread, and to inform more rapid and effective diagnostic and therapeutic strategies.

## Background

*Mycobacterium tuberculosis* is the causative agent of tuberculosis (TB), which is most often spread person-to-person via cough aerosols. Although many individuals who are exposed to *M. tuberculosis* never develop active disease, the World Health Organization (WHO) estimated 10 million new cases of active TB and 1.3 million deaths in 2017 alone [1].

Since its initial documentation in the 1940s [2], drug-resistant TB has threatened public health control efforts. In 2016, there were an estimated 490,000 new cases of multidrug-resistant (MDR) TB, which is defined by phenotypic resistance to both isoniazid and rifampicin [3]. Approximately 10% of MDR-TB cases globally can be classified as extensively drug-resistant (XDR), indicating that there is concomitant resistance to quinolones (such as the fluoroquinolones, levofloxacin, and moxifloxacin) and to a second-line injectable agent (amikacin, kanamycin, or capreomycin) [3]. As expected, drug-resistance patterns predict treatment outcome; in 2015, TB treatment success overall was 83%, whereas the success rate was 54% for MDR-TB or rifampicin-resistant-TB (RR-TB) and only 30% for XDR-TB [4].

Culture-based techniques remain the current reference standard for both diagnosis and drug-susceptibility testing of TB, but these processes are time-consuming and require specialized laboratory capacity. More recently, the use of rapid molecular tests for the diagnosis of TB has increased globally, particularly the use of Xpert MTB/RIF (Cepheid, Sunnyvale, CA), a PCR-based assay that simultaneously detects the presence of *M. tuberculosis* and resistance to rifampicin.

Current recommendations for the treatment of drug-susceptible TB include a 6-month course of a multi-drug regimen of rifampicin, isoniazid, pyrazinamide, and ethambutol. Historically, treatment of MDR- or XDR-TB involved the long-term use of second-line drugs, including injectable agents [5]. More recently, the MDR-TB treatment landscape has changed with the introduction of multiple novel second-line drugs that can be administered orally (Table 1). In 2012, bedaquiline, a diarylquinolone, became the first TB drug from a novel drug class to receive US Food and Drug Administration (FDA) approval in over 40 years [48, 49] (Table 1). Another oral agent, delamanid, a nitro-dihydro-imidazooxazole derivative, has also shown promise for TB treatment [50, 51].

**Table 1:** Antitubercular drug-resistance mechanisms^a^

WHO category	Drug or drug class	Resistance genes	Rv number	Gene function	Mechanism of drug resistance	Reference(s)
First-line agents	Rifamycins (for example, rifampicin)	*rpoB*	Rv0667	RNA polymerase	Target modification	[6]
		*ponA1*	Rv0050	Probable bifunctional penicillin-binding protein	Unknown	[7]
	Isoniazid	*katG*	Rv1908c	Catalase-peroxidase enzyme	Decreased drug activation	[8]
		*inhA*	Rv1484	NADH-dependent enoyl-acyl carrier protein	Target amplification or modification	[9, 10]
	Pyrazinamide^b^	*pncA*	Rv2043c	Pyrazinamidase	Decreased drug activation	[11, 12]
		*panD*	Rv3601c	Aspartate decarboxylase	Unknown	[13]
		*rpsA*	RRv1630	Ribosomal protein S1	Target modification	[14]
	Ethambutol^b^	*embCAB* operon	Rv3793-5	Arabinosyltransferase	Target modification	[15, 16]
		*ubiA*	Rv3806c	Arabinogalactan synthesis	Gain-of-function	[15]
Group A	Levofloxacin Moxifloxacin	*gyrA*	Rv0006	DNA gyrase A	Target modification	[17, 18]
		*gyrB*	Rv0005	DNA gyrase B	Target modification	[18]
	Bedaquiline	*atpE*	Rv1305	ATP synthase	Target modification	[19]
		*pepQ*	Rv2535c	Putative Xaa-Pro aminopeptidase	Unknown	[20]
		Rv0678	Rv0678	Transcriptional regulator of *mmpL5*	Drug efflux	[21, 22]
	Linezolid	*Rrl*	NA	23S rRNA	Target modification	[23]
		*rplC*	Rv0701	50S ribosomal protein L3	Target modification	[24]
Group B	Clofazimine	*pepQ*	Rv2535c	Putative Xaa-Pro aminopeptidase	Drug efflux	[20]
		Rv0678	Rv0678	Transcriptional regulator of *mmpL5*	Drug efflux	[21]
	Cycloserine Terizidone	*Ald*	Rv2780	L-alanine dehydrogenase	Substrate shunting	[25]
		*alr*	Rv3423c	Alanine racemase	Target modification	[26, 27]
		*ddl*	Rv2981c	D-alanine-D-alanine ligase	Target modification	[27]
		*cycA*	Rv1704c	Bacterial D-serine/L-and D-alanine/glycine/D-cycloserine proton symporter	Mechanism not confirmed	[28]
Group C	Delamanid Pretomanid	*ddn*	Rv3547	Oxidative stress	Decreased drug activation	[29]
		*fgd1*	Rv0407	Glucose-6-phosphate oxidation	Decreased drug activation	[29]
	Imipenem/cilastatin	*crfA*	Rv2421c-Rv2422 intergenic	Unknown	Drug inactivation	[30]
	Amikacin, Capreomycin, Kanamycin^c^	*Rrs*	NA	16S rRNA	Target modification	[31]
	Streptomycin	*rpsL*	Rv0682	12S ribosomal protein	Target modification	[32–35]
		*rrs*	NA	16S rRNA	Target modification	[36]
		*gidB*	Rv3919c	7-Methylguanosine methyltransferase	Target modification	[37]
	Ethionamide Prothionamide	*ethA*	Rv3854c	Mono-oxygenase	Decreased drug activation	[38, 39]
		*ethR*	Rv3855	Transcriptional regulatory repressor protein (TetR)	Decreased drug activation	[39]
		*inhA*	Rv1484	NADH-dependent enoyl-acyl carrier protein	Target amplification or modification	[10]
	Para-aminosalicylic acid (PAS)	*folC*	Rv2447c	Folate pathway	Decreased drug activation	[40]
		*dfrA*	Rv2763c	Dihydrofolate reductase	Target amplification	[40]
		*thyA*	Rv2764c	Thymidylate synthase	Target modification	[41, 42]
		*thyX*	Rv2754c	Catalyzes dTMP and tetrahydrofolate	Mitigating target inhibition	[43]
		*ribD*	Rv2671	Enzyme in riboflavin biosynthesis	Mitigating target inhibition	[40, 44]
Other medicines^c^	Kanamycin	*Eis*	Rv2416c	Aminoglycoside acetyltransferase	Inactivating mutation	[45]
	Capreomycin	*tlyA*	Rv1694	rRNA methyltransferase	Target modification	[46]

*Abbreviations*: *MDR-TB* multidrug-resistant tuberculosis, *NA* not applicable, *RR-TB* rifampicin-resistant tuberculosis, *WHO* World Health Organization

^a^Antitubercular drugs are listed by the 2018 WHO grouping of medicines recommended for use in longer, individualized MDR-TB regimens [47]. For each drug or drug class, the specific genes in which drug-resistance mutations are commonly identified are listed with their gene name, gene number (Rv number), gene function, and the confirmed or putative mechanisms of resistance. ^**b**^Pyrazinamide and ethambutol are first-line TB drugs that also are categorized as Group C medicines for the treatment of longer MDR-TB regimens. ^c^Kanamycin and capreomycin are no longer recommended to be included in longer, individualized MDR/RR-TB regimens

In 2018, the WHO published updated treatment guidelines for MDR/RR-TB [47], recommending fully oral MDR regimens for many patient groups. Recommended treatment strategies include both shorter, standardized MDR regimens (for 9 to 12 months) and longer, individualized treatment regimens (for 18 to 20 months). The updated guidelines group antitubercular drugs on the basis of how they should be combined to create individualized, longer MDR-TB regimens [47] (Table 1).

Despite advances in both diagnostics and therapeutics for TB, challenges remain. Obstacles for rapid *M. tuberculosis* diagnosis include: (i) the imperfect sensitivity of molecular tests for the detection of this pathogen, particularly in the case of paucibacillary TB (where there is a lower bacterial burden); (ii) lack of comprehensive molecular assays due to incomplete knowledge of all resistance mutations in TB; and (iii) technical limitations to the numbers of mutations that can be included on diagnostic molecular platforms. Furthermore, the rollout of rapid diagnostic platforms to low-resource settings has been a challenge. Remaining treatment challenges include: prolonged treatment courses, leading to greater drug exposure, toxicity, and non-compliance; unacceptable side-effect profiles; logistics of drug access; and re-infection [52].

The dawning of the new age of genome sequencing began to revolutionize our approach to human diseases, including TB. In 1998, Cole et al. [53] reported the complete genome sequence of the *M. tuberculosis* reference strain H37Rv, which was approximately 4.41 million base pairs in length and encoded approximately 4000 genes. The first sequencing of a clinical reference strain, CDC1551, quickly followed [54]. An accompanying editorial optimistically stated: “After several decades in the slow lane of classical microbiology, *M. tuberculosis* is once again at the cutting edge of science” [55]. However, even at the time of these breakthroughs, there was recognition that translating these genomic data into clinical benefit would prove challenging [55]. Despite these challenges, it is clear, more than 20 years later, that *M. tuberculosis* genomic data have been remarkably useful in improving our understanding of how drug-resistant TB evolves and spreads and in helping to inform diagnostics and therapies.

In this review, we discuss the molecular epidemiologic and diagnostic advances made by sequencing *M. tuberculosis*, with a focus on drug-resistant TB. We do not review the practice of whole-genome sequencing (WGS) of *M. tuberculosis* as this has been reviewed recently [56]. Key findings that are discussed include the use of WGS to identify drug-resistance determinants in *M. tuberculosis* and to elucidate the evolution and spread of drug-resistant organisms, and the clinical applications of this technology (Table 2).

**Table 2:** Spotlight on whole-genome sequencing studies of drug-resistant *M. tuberculosis*

Reference	Description	Advances
Identifying *M. tuberculosis* drug-resistance determinants		
Farhat et al. 2013 [7]	Large-scale WGS project: sequencing of 116 genomes from around the globe	Developed a phylogenetic convergence test, PhyC, to identify resistance associations; validated *ponA1* mutations that increase MIC for rifampicin
Zhang et al. 2013 [57]	Large-scale WGS project: sequencing of 161 genomes from China	Identified genes that are under positive selection and have increased mutation frequencies in drug-resistant isolates
Walker et al. 2015 [58]	Analysis of 23 candidate resistance genes from 3651 clinical isolates	Demonstrated that drug-resistance in *M. tuberculosis* can be predicted with high sensitivity and specificity
Desjardins et al. 2016 [25]	Use of a combination of the correlated evolution test and a GWAS framework to identify drug-resistance-associated mutations in 498 genomes from China and South Africa	Identified *ald* loss-of-function as a novel mechanism of D-cycloserine resistance
Coll et al. 2018 [59]	GWAS study of 6465 *M. tuberculosis* clinical isolates from more than 30 countries	Identified new resistance-associated mutations in *ethA* and the *thyX* promoter
The Cryptic Consortium and the 100,000 Genomes Project [60]	Prediction of first-line-drug susceptibility in a dataset of 10,209 clinical isolates from 16 countries	Predicted drug-susceptibility phenotypes with high sensitivity and specificity using WGS in a large global dataset
Within-patient evolution of resistance		
Eldholm et al. 2014 [61]	WGS of nine serial isolates cultured from a single patient over a 42-month period	First documented case of the evolution of susceptible TB into XDR-TB in a single patient in response to selective drug pressure
Trauner et al. 2017 [62]	Very deep WGS of serial sputum specimens from patients receiving treatment for TB	Demonstrated that the combination of multiple active drugs prevented fixing and dominance of transient mutants. The fewer drugs used, the more likely it was that resistance would develop and become fixed
Transmission versus de novo evolution of resistance		
Nikolayevskyy et al. 2016 [63]	Literature review including meta-analysis of 12 studies published between 2005 and 2014	Showed that WGS studies have higher discriminatory power than fingerprinting techniques and can more sensitively detect transmission events
Ioerger et al. 2010 [64]	WGS of 14 phenotypically diverse strains within the Beijing lineage in South Africa	Showed that resistance mutations arose independently multiple times, and that XDR-TB isolates may be less fit and less able to transmit
Shah et al. 2017 [65]	Sequencing of more than 400 strains from South Africa	The majority of cases of XDR-TB in KwaZulu-Natal were due to transmission rather than de novo evolution
Manson et al. 2017 [66]	WGS of a set of 5310 isolates, with diverse geographical origin, genetic background, and drug-resistance profiles	Demonstrated that both de novo evolution and transmission contribute to drug-resistance worldwide
Geographic spread of multidrug-resistance		
Cohen et al. 2019 [67]	Further analysis of geographic trends in MDR strains within the set of 5310 strains from Manson et al. [66]	Revealed extensive worldwide spread of MDR-TB clades between countries of varying TB burden
Nelson et al. 2018 [68]	Sequencing of 344 patients with XDR-TB, combined with global positioning system coordinates	Identified many cases of probable person-to-person transmission (≤ 5 SNPs) between people living a median of 108 km apart, suggesting that drivers of XDR-TB transmission include migration between urban and rural areas
Order of acquisition of resistance mutations		
Cohen et al. 2015 [69]	WGS and drug-susceptibility testing on 337 clinical isolates collected in Kwazulu-Natal, South Africa	Showed that stepwise accumulation of mutations leading to XDR-TB in Kwazulu-Natal occurred over decades. Established the order of acquisition of drug-resistance mutations leading to XDR-TB, showing that isoniazid resistance almost always evolved prior to rifampicin resistance
Eldholm et al. 2015 [70]	WGS of all 252 available clinical isolates from an outbreak in Argentina	Showed stepwise accumulation of mutations leading to the development of MDR-TB in Argentina
Manson et al. 2017 [66]	WGS of 5310 isolates with diverse geographical origin, genetic background, and drug-resistance profiles	Established that a clear order of acquisition of resistance mutations holds globally: isoniazid resistance overwhelmingly evolves prior to rifampicin resistance across all geographies, lineages, and all time periods (including decades after rifampicin introduction)
Evolution of compensatory and stepping-stone mutations		
Fonseca et al. 2015 [71]	Review paper	Discussed the evolution of compensatory mutations that can ease fitness effects caused by resistance
Comas et al. 2012 [72]	Comparison of the genome sequences of ten clinical rifampicin-resistant isolates to those of the corresponding rifampicin-susceptible isolates from the same individual at an earlier timepoint	Identified compensatory mutations in *rpoB* that conferred high competitive fitness in vitro and were also found frequently in clinical populations
Casali et al. 2014 [73]	Large-scale analysis of 1000 strains from Russia	Examined strains with primary rifampicin-resistance mutations in *rpoB*, and identified accompanying compensatory mutations in *rpoA* and *rpoC*
Cohen et al. 2015 [69]	WGS and drug-susceptibility testing of 337 clinical isolates collected in Kwazulu-Natal, South Africa	Identified putative rifampicin compensatory mutations in *rpoA*, *rpoB*, and *rpoC*
Merker et al. 2018 [74]	Sequencing of highly resistant TB strains from Central Asia	Showed that the presence of rifampicin compensatory mutations are associated with transmission success and higher drug-resistance rates
Coll et al. 2018 [59]	GWAS study of 6465 *M. tuberculosis* clinical isolates from more than 30 countries	Identified putative compensatory mutations for pyrazinamide and PAS resistance
Safi et al. 2018 [15]	Genetically and biochemically characterized strains selected in vitro for ethambutol resistance	Showed that multi-step selection is required to achieve the highest levels of ethambutol resistance
Understanding mixed infections and spatial heterogeneity within a patient		
Köser et al. 2013 [75]	WGS for rapid drug-susceptibility testing of a patient with XDR-TB	Determined that the patient carried two different XDR-TB Beijing strains with differing resistance mutations
Liu et al. 2015 [76]	Deep WGS of serial sputum isolates within a patient	Identified three dominant subclones differing by 10–14 SNPs within a single patient, with different resistance patterns and probably different anatomical distributions
Lieberman et al. 2016 [77]	Sequencing of samples from post-mortem biopsies from different body sites	Observed sublineages evolving within a patient, as well as distinct strains from mixed infections that were differentially distributed across body sites
Dheda et al. 2018 [78]	Sequencing of samples biopsied from seven different body sites, as well as pre-treatment and serial sputum samples	Showed that drug concentrations at different sites were inversely correlated with bacterial MICs. Sequencing and comparison to sputum samples suggested ongoing acquired resistance
Sobkowiak et al. 2018 [79]	Assessed methods for detecting mixed infections using WGS data from in vitro and in silico artificially mixed *M. tuberculosis* samples	Frequency of mixed infections in the Karonga Study in Mali is approximately 10% and only associated with year of diagnosis, not with age, sex, HIV or prior TB infection. Computational methods can identify mixed infections using WGS data
Bench to bedside with WGS		
Pankhurst et al. 2016 [80]	Prospective study evaluating the use of WGS for diagnosis	Compared WGS of positive liquid cultures to routine laboratory workflows. Illumina MiSeq-based bioinformatics classification of species and drug resistance was faster (by a median of 21 days) and cheaper (by 7%), yet offered similar accuracy to routine techniques
Doughty et al. 2014 [81]	Sequencing-based detection without culturing	Proof-of-concept culture-free metagenomics detection of *M. tuberculosis* from sputum samples using Illumina MiSeq
Votintseva et al. 2017 [82]	Evaluation of Oxford Nanopore sequencing for diagnostic or surveillance purposes	Proof-of-concept detection of *M. tuberculosis* DNA in sputum samples using a portable sequencer

*Abbreviations*: *GWAS* genome-wide association study, *MDR* multidrug-resistant, *MIC* minimum inhibitory concentration, *PAS* para-aminosalicylic acid, *SNP* single nucleotide polymorphism, *TB* tuberculosis, *XDR* extensively drug-resistant

## Identifying *M. tuberculosis* drug-resistance determinants

Drug resistance in *M. tuberculosis* is the result of chromosomal mutations in existing genes that are passed along through vertical descent, that is, passed from mother to daughter cells. Unlike many other bacterial pathogens, *M. tuberculosis* rarely recombines via lateral exchange of DNA [83] and also lacks plasmids. Many of the resistance determinants were discovered before the sequencing of the *M. tuberculosis* genome was completed. By 1998, resistance mechanisms had already been discovered for classical first- and second-line TB drugs including isoniazid (alterations in genes *katG* and *inhA*); rifampicin (in *rpoB*); streptomycin (in *rrs* and *rpsL*); pyrazinamide (in *pncA*); ethambutol (in *embB*); quinolones (in *gyrA*); and kanamycin (in *rrs*) (reviewed in Ramaswamy and Musser [84]) (Table 1). However, the targeted amplification and sequencing of known or suspected resistance genes revealed that these mechanisms were insufficient to explain all phenotypic resistance [85, 86], and resistance mechanisms for several newer drugs—including pretomanid, bedaquiline, and delamanid—were discovered over the next eight years during a period when WGS was becoming routine. Together, in the past 20 years, WGS-based approaches, focused on both laboratory-derived and naturally circulating populations of drug-resistant *M. tuberculosis*, have provided a more complete account of the genomic features that cause treatment resistance, enabling the identification of novel resistance mechanisms for existing drugs, and the determination of the mechanisms of action of newly discovered drugs.

### Identifying resistance determinants in laboratory-derived mutants

Drug-resistant mutants can be derived in vitro by growing drug-susceptible *M. tuberculosis* strains in drug-containing media, and selecting for mutants that are able to grow in the presence of the drug. Sequencing laboratory-derived resistant mutants has played a critical role in identifying both the mechanism of action of new TB drug classes, including diarylquinolines (for example, bedaquiline) [19] and nitroimidazopyrans (for example, PA-824) [19, 29], and rare resistance mechanisms for established antitubercular drugs, including ethambutol [15], pyrazinamide [13], carbapenems [30], cycloserine [87], clofazimine, and bedaquiline [20]. For example, WGS of laboratory mutants identified drug efflux as a mechanism of resistance to clofazimine and bedaquiline [20–22], and this approach continues to be a mainstay for identifying the mechanism of action of compounds that are in development for TB [88].

Although laboratory-derived mutants are helpful in elucidating novel resistance mechanisms, mutations that have evolved in laboratory settings do not always match those in clinical isolates of drug-resistant *M. tuberculosis* [89, 90], for reasons that are largely unknown. Studies by Ford et al. [91, 92] suggested that these mismatches could not be explained by differences in the mutation rate in these settings, because the in vitro mutation rate of *M. tuberculosis* correlates well with the in vivo mutation rate in both humans and in a macaque model. Differences in the relative fitness of specific mutants grown in in vitro compared to in vivo conditions are probably responsible for these mismatches, but more work is needed. Regardless of the reason, if the goal is to identify a full complement of resistance mutations on which to base molecular diagnostics, isolates from clinical collections must be studied as these bacteria have evolved their resistance within the host.

### Quantifying and identifying resistance determinants in clinical strains

Among the larger studies exploring resistance in natural populations, Walker et al. [58] analyzed the genomes of 3651 drug-resistant and -susceptible *M. tuberculosis* isolates for associations between phenotypic resistance to eight first- and second-line drugs, and then predicted genotypic resistance on the basis of a compiled catalog of 232 resistance mutations in 23 candidate resistance genes. Resistance to most drugs could be predicted accurately, with a mean sensitivity of 92% and specificity of 98%, suggesting that the majority of resistance—particularly for first-line drugs—is explained by known mechanisms and mutations (Table 1). Numerous other studies have found similar results using smaller datasets [7, 25, 57, 69, 93, 94]. This result was echoed in a more recent study by the Comprehensive Resistance Prediction for Tuberculosis (CRYPTIC) Consortium and the 100,000 Genomes Project that focused solely on first-line drugs, which included analysis of 10,209 globally diverse *M. tuberculosis* isolate genomes against a database of mutations identified in a literature search [60]. Notably, predictions for mutations that are associated with resistance to pyrazinamide were greatly improved over earlier predictions; this study achieved 91.3% sensitivity in predicting resistance to this drug compared to 57% sensitivity in Walker et al. [58]. Although the news has been encouraging with respect to completing the catalog of mutations that cause resistance to first-line drugs, few studies have attempted to predict resistance to second-line drugs [95]. Some of these drugs, such as D-cycloserine, pyrazinamide, and para-aminosalicylic acid (PAS), are more difficult to assay because they have been reported to have variable drug phenotypes in clinical microbiology laboratories [96] (discussed later).

To fill gaps in the catalog of drug-resistance mechanisms, genome-wide association study (GWAS) approaches, originally designed for use on human genomic data, have been adapted for non-recombining microbes such as *M. tuberculosis* and used to predict novel resistance mechanisms [97, 98] (Table 3). The majority of GWAS predictions remain experimentally unverified, but several new resistance-associated genotypes have been validated. Farhat et al. [7] sequenced 116 *M. tuberculosis* genomes and developed a phylogenetic convergence test, ‘PhyC’, to identify resistance associations. They identified a mutation in *ponA1* (c.1095G>T) and showed that it conferred a minimum inhibitory concentration (MIC) to rifampicin that was twofold higher than that of wild-type bacteria. Zhang et al. [57] sequenced 161 genomes from China and searched for genes that appeared to be under positive selection and more frequently mutated in drug-resistant isolates. Resistance-associated polymorphisms in two intergenic regions upstream of the known resistance genes *thyA*-Rv2765 and *thyX*-*hsdS*.1 were shown to cause increased gene expression of a *lacZ* construct in *Mycobacterium smegmatis*, suggesting that these mutations may mediate PAS resistance through the overexpression of downstream genes.

**Table 3:** Publicly available software packages implementing microbial GWAS methods for identifying drug-resistance-associated genetic variants in bacteria

Method	Details of approach	Key recent studies and advances achieved in identifying drug-resistance-associated genetic variants	Availability	Reference(s)
bugwas	Uses linear mixed models with a correction for population stratification. Uses SNPs identified through mapping to a reference	Applied to identify resistance to 17 drugs across 3144 isolates from four diverse species of bacteria, including *M. tuberculosis* [99]. Confirmed that some major known resistance determinants could be recovered. The method was recently extended in a kmer-based method based on bugwas [100]	https://github.com/sgearle/bugwas	[99, 100]
SEER	Uses logistic and linear regression with a correction for population stratification. Uses SNPs identified through mapping to a reference	Initially applied to *Streptococcus*. To date, has not been applied to *M. tuberculosis*	https://github.com/johnlees/seer/wiki	[101]
treeWAS	Uses a phylogenetic test to identify convergent evolution using kmers, which can detect both individual variants and gene presence or absence agnostic of a reference	Initially applied to *Neisseria meningitidis*. Has not yet been applied to *M. tuberculosis*	https://github.com/caitiecollins/treeWAS	[102, 103]
phyC	Uses phylogenetic tests to identify convergent evolution, using SNPs identified through mapping to a reference	Identified 39 genomic regions that are potentially involved in resistance, and confirmed a rifampicin-conferring mutation in *ponA1* [7]. Used within a mixed-regression framework to detect resistance determinants to 14 drugs in a dataset of 6465 global clinical isolates. Identified new ethionamide-resistance codons in *ethA* and PAS-resistance mutations in the thyX promoter [59]	https://bitbucket.org/rpetit3/visa-gwas	[7, 59, 102]

*Abbreviation*: *GWAS* genome-wide association study, *SNP* single nucleotide polymorphism

Desjardins et al. [25] used a combination of the correlated evolution test [104] (to test for correlated evolution of genotype and phenotype) and a simple GWAS framework to search for novel drug-resistance mechanisms in 498 genomes from South Africa and China. Of note, they combined all variants within each gene that were predicted to inactivate gene function, and used these combinations as the input into the association test to increase statistical power in the detection of genomic features that are associated with resistance. They found that loss-of-function mutations in *ald* (Rv2780), which is predicted to encode an alanine dehydrogenase, correlated with unexplained resistance [25]. They also confirmed experimentally that these mutations conferred increased resistance of laboratory and clinical isolates to D-cycloserine [25], a key drug in MDR- and XDR-TB regimens that has severe psychiatric and central nervous system toxicities.

Hicks et al. [105] used the algorithm phyOverlap to perform a GWAS on 549 clinical isolates from China, in which they identified mutations that disproportionately occurred in isoniazid-resistant isolates. In addition to known resistance and compensatory mutations for first- and second-line drugs, they identified an association with *prpR* (Rv1129c). They then went on to characterize *prpR* as a transcriptional regulator of propionate metabolism which, instead of drug resistance, confers tolerance to multiple antibiotics in a macrophage model of infection.

In one of the largest GWAS published to date, Coll et al. [59] combined PhyC with a GWAS approach within a mixed-regression framework to detect determinants of resistance to 14 drugs in a large dataset of 6465 global *M. tuberculosis* clinical isolates. Although no functional experiments were performed to validate the predictions, new resistance-associated mutations were identified, including new codons in *ethA* (a gene known to activate ethionamide, which is a prodrug) that are associated with ethionamide resistance, and mutations in the *thyX* promoter associated with PAS resistance. Mutations in the promoter of *thyX* have been previously shown to upregulate *thyX* [43, 57, 106].

### Predicting susceptibility and drug resistance in M. tuberculosis

As the list of suspected resistance determinants grows, there has been a need to establish well-curated databases of drug-resistance single nucleotide polymorphisms (SNPs) [107]. Initially, SNP databases, including TBDB [108] and PATRIC [109], were created to bring together genome annotation data and other functional data. Unfortunately, some of the pioneering databases of drug-resistance-associated mutations in *M. tuberculosis*, including TBDReamDB [110], have not been maintained to include emerging data.

Software and web-based tools have also been developed to enable the community to infer TB drug resistance from WGS data. These tools include CASTB [111], KVarQ [112], MyKrobe Predictor TB [113], PhyResSE [114], TBProfiler [115], and TGS-TB [116]. Studies have compared the sensitivity and specificity of these tools in predicting drug resistance [117–119], and have found that they tend to perform quite well for first-line drugs but less optimally for second-line drugs. In addition to tools, there have been improvements to databases, including the creation of the Relational Sequencing TB Database Platform (ReSeqTB) [120, 121] and efforts from the CRyPTIC Consortium [122], which seeks to develop a curated database of clinically relevant drug-resistance mutations.

Continued refinement of these drug-resistance databases and prediction tools is necessary. Miotto et al. [123] performed a systematic review in which they assigned a confidence level to associations of individual and groups of mutations with phenotypic drug resistance. Importantly, they identified that certain mutations that are included in current commercial diagnostic tests, including *eis* c-2a, do not have a convincing association with drug resistance. Input from ongoing large sequencing projects will be needed to optimize the inference of resistance phenotypes from sequence data, especially for mutations that are present at low frequency in natural populations.

### Challenges in uncovering the remaining resistance elements

Although WGS approaches have been successful in identifying resistance mechanisms, there are computational and experimental challenges that hamper efforts to complete the catalog of TB drug resistance. For example, for non-recombining organisms such as *M. tuberculosis*, interpretation of GWAS output can be complicated because non-causal variation can be tightly linked to causal variation [124]. Furthermore, as a result of frequent multidrug resistance, resistance mutations for one drug can appear to be highly associated with phenotypic resistance to multiple drugs [25], and confirmatory wet lab studies, which are non-trivial in *M. tuberculosis*, are often necessary to identify causal resistance mutations correctly. In addition, genotype–phenotype associations are largely dependent on accurate phylogenies, and phylogenetic reconstruction can be challenging in *M. tuberculosis* because of its slow rate of evolution [92, 125–128], which gives rise to relatively few SNPs in clinical isolates.

When defining phenotypic resistance, different studies often use different drug concentration cutoffs and test in different media, complicating the meta-analysis of multiple datasets. In addition, phenotypic resistance testing of some antitubercular drugs, including pyrazinamide and D-cycloserine, is notoriously challenging and unreliable [129], introducing phenotypic inaccuracies that can confound analyses. Furthermore, the dichotomous classification of phenotypic resistance as ‘resistant’ or ‘susceptible’ will fail to identify drug-resistance mutations that result only in minimal increases in MIC, and there is emerging evidence that such mutations may be clinically relevant. TB relapse following treatment has been found to occur more commonly in individuals who harbored *M. tuberculosis* isolates that were susceptible to, yet had minimally increased MIC values for, either isoniazid or rifampicin [130]. Future study designs that address phenotypic resistance as a spectrum, rather than a binary value, will be needed to identify such mutations.

Heteroresistance, defined as the coexistence of pathogen populations that have differing nucleotides at a specific drug-resistance locus [131], can also confound genotype–phenotype comparisons [132–134]. A bacterial culture in which only a small fraction of the population is resistant can appear to be resistant when tested on media containing a drug, yet when grown on drug-free media for genome sequencing, the sensitive fraction can dominate, resulting in a genotypic prediction of sensitivity [132]. The problem of heteroresistance seems to be particularly common with fluoroquinolone resistance [135].

Last, innate characteristics of the *M. tuberculosis* genome—namely, highly repetitive DNA sequences and the high guanine-cytosine (GC) content of the genome (65.6%) [53]—present technical difficulties for both WGS and bioinformatic analyses. GC-rich regions can be troublesome for library PCR amplification and sequencing, and reads that represent highly repetitive regions of the genome can confound alignments by mapping to multiple regions of the genome and hampering accurate de novo assemblies. In addition, approximately 10% of the coding regions in *M. tuberculosis* are dedicated to two repetitive protein families that are unique to mycobacteria (the PE and PPE families), which have conserved Pro-Glu (PE) and Pro-Pro-Glu (PPE) motifs [53]. Even with WGS investigation [136], the function of the PE and PPE genes has remained elusive, although recent studies have suggested that they may play a role in virulence [137]. Their association with drug resistance remains largely unexplored because bioinformatic studies of *M. tuberculosis* often exclude these genes [138, 139]. In the future, long-read sequencing technology may allow these regions to be sequenced successfully in order to assess if they have a role in drug resistance.

## Understanding the evolution and spread of drug resistance in *M. tuberculosis*

Prior to WGS, the diversity and epidemiology of resistant *M. tuberculosis* were examined using DNA fingerprinting techniques, like IS6110 restriction fragment length polymorphism (RFLP) typing [140], spoligotyping (spacer oligonucleotide typing, a method of typing strains according to the distinct hybridization patterns of their spacer oligonucleotides) [141], and mycobacterial interspersed repetitive units-variable number of tandem repeats (MIRU-VNTR) typing [142–145]. These techniques enabled assessments of the diversity of resistant strains in specific geographic regions [146–149] and, when combined with the genetic profiling of resistance mutations, allowed strain-level monitoring of patients on TB therapy [150].

The dramatic increase in resolution afforded by WGS has extended the sensitivity and resolution with which the diversity and evolution of drug-resistant *M. tuberculosis* can be assessed. This has resulted in the more confident identification of cases of recent transmission [151] and re-infection [152], and has provided insights into the evolution of resistance within individual patients and across populations. WGS has also enabled more sensitive differentiation of de novo acquisition of resistance (where resistance mutations emerge within a host) from person-to-person transmission of resistance, a critical capability given that these two scenarios require different health-system responses in order to stem resistance.

### Within patient evolution of drug resistance

Despite the slow evolutionary rate of *M. tuberculosis*, estimated at 0.3–0.6 SNPs/genome/year [69, 92, 125–128], experimental data suggest that drug resistance can evolve within an individual patient during TB treatment. Eldholm et al. [61] described the first documented case of XDR evolution of *M. tuberculosis* from a fully susceptible ancestor within a single patient, by sequencing nine serial isolates collected over a 42-month period. During this time, seven known resistance mutations emerged in a stepwise fashion after the clinical use of each corresponding drug, revealing how TB drug pressures can rapidly shape *M. tuberculosis* populations in vivo.

However, the evolution of drug resistance within a host is not always linear, and instead can involve a complex interplay of heterogeneous *M. tuberculosis* populations [153, 154]. In particular, transient genetic diversity can exist before a dominant clone emerges. In addition, as the size of the transmission bottleneck (the number of bacteria transmitted during an infection event) in *M. tuberculosis* is not well understood [155], it is difficult to estimate the relative contribution of diversity that is transmitted to the patient versus diversity that evolves within the patient. Numerous WGS studies, performed either on isolates or directly on DNA extracted from serially collected sputum samples, have revealed substantial transient genetic diversity in pathogen populations within patients, particularly within resistance genes [61, 62, 106, 156–159]. This diversity was observed to endure months before a single variant became fixed in the population (the situation when only a single variant remains). In the study by Eldholm et al. [61] mentioned above, the seven resistance-conferring mutations that eventually dominated were from amongst 35 mutations observed in total throughout the sampling period [61, 160]. They joined eight other mutations that were not resistance-associated but that also became fixed in the population, probably as the result of a phenomenon called ‘hitchhiking’ in which non-adaptive mutations are selected because of their linkage and physical proximity to consequential mutations.

The relative fitness cost of drug-resistance mutations often determines which mutations become fixed within a host. While multiple mutations that confer resistance to a specific drug can evolve repeatedly, mutations conferring no or little fitness cost are typically selected, resulting in fixed dominant mutations [61, 156]. Compensatory mutations (discussed in more detail later), which serve to counterbalance the deleterious effects of acquired resistance, have also been shown to emerge during treatment [156].

WGS has also revealed how combination chemotherapy effectively prevents the emergence of drug resistance during treatment for TB. In a study of very deep WGS of serial sputum specimens from patients receiving treatment for TB, Trauner et al. [62] demonstrated that the combined action of multiple active drugs prevented transient mutants from fixing within a population and becoming dominant. The fewer the drugs that were applied, the more likely it was that resistance would develop and become fixed.

### Population views of drug-resistance evolution

A number of careful WGS studies have empirically established SNP-based criteria to discriminate cases of recent transmission from unrelated infections—usually using the criterion that recently transmitted strains differ by fewer than 6–12 total SNPs across the *M. tuberculosis* genome [63, 125, 126, 161]. In a 2016 review, Nikolayevskyy and colleagues [63] systematically compared WGS to fingerprinting techniques for detecting transmission, including a meta-analysis of 12 studies published between 2005 and 2014. They concluded that results from WGS studies not only have higher discriminatory power, but they also enable more sensitive detection of transmission events that may have been missed by epidemiologic methods.

Although traditional spoligotyping analyses suggested that drug-resistant strains were diverse, WGS of clinical isolates began to reveal the full breadth of diversity in resistant *M. tuberculosis*. The TB epidemic in South Africa over the past two decades has been well-studied in this regard. In an early WGS investigation, Ioerger et al. [64] examined 14 phenotypically diverse strains within the Beijing lineage and showed that resistance mutations arose independently multiple times, and that XDR isolates may be less fit and less able to transmit. WGS studies across larger sets of strains from the same region in South Africa suggested that, although de novo resistance is indeed common, highly resistant strains (including MDR and XDR strains) have the ability to spread broadly by person-to-person transmission. This includes the ongoing transmission of a circulating XDR clone in South Africa that is linked to the infamous Tugela Ferry XDR outbreak [162] that brought XDR-TB to the world stage in 2005. A more recent large-scale study confirmed that XDR strains have been broadly transmitted person-to-person in KwaZulu-Natal [65].

The patterns observed in South Africa hold for many other parts of the world. Recent studies have shown that patterns of both de novo evolution and person-to-person spread of drug resistance in *M. tuberculosis* also occur in Belarus, Russia, England, and Malawi [73, 139, 159, 163, 164]. In a composite analysis of over 5000 *M. tuberculosis* isolates from patients from around the globe, Manson et al. [66] confirmed that both de novo evolution and person-to-person transmission are important factors for the rise and spread of drug-resistant TB worldwide. The emergence of MDR and XDR *M. tuberculosis* was found to be a frequent occurrence that is distributed fairly evenly across the globe [66]. This analysis also predicted that 37% of MDR isolates in this study had spread person-to-person, which is probably a vast underestimate of how frequently MDR is transmitted once evolved [66].

Geographic movement of people is also an important consideration with regard to person-to-person transmission. Further examination of the MDR clades from Manson et al. [66] revealed that they included widespread international, and even intercontinental, dissemination of strains that were separated by as few as four SNPs, probably due to spread via international travel [67]. Even within a single province in South Africa, Nelson et al. [68] showed, using genomic sequence data and global positioning system coordinates, that many cases of person-to-person transmission (with ≤ 5 SNPs) of XDR-TB occur between people living a median of 108 km apart, pointing to migration between urban and rural areas as a driver of TB spread. Collectively, these studies reinforce the idea that the geographic movement of people must be taken into consideration in any strategy for controlling the spread of TB resistance.

### Ordering of the acquisition of resistance and compensatory mutations

Recent WGS studies have helped to illuminate the steps or ‘fitness landscape’ through which *M. tuberculosis* develops and compensates for drug resistance. Several studies [66, 69, 70] have shown that the order of acquisition of drug-resistance mutations in complex resistance cases is partly constrained in clinical *M. tuberculosis*. For example, in MDR-TB, isoniazid resistance (most often involving a *katG* S315T mutation) overwhelmingly evolves prior to resistance to rifampicin and second-line drugs. This was first shown using regional datasets from South Africa [69] and Argentina [70], and recently confirmed by Manson et al. [66] using a global dataset of 5310 strains. In the study by Manson et al. [66], this ordering was shown to hold true over 95% of the time, even for distinct global regions and time frames, including times when both rifampicin and isoniazid were in use, suggesting that the earlier introduction of isoniazid in the 1950s was not the major contributor to this effect. It was also shown that *inhA* promoter mutations that confer isoniazid resistance (such as those observed by Perdigão et al. [165] in Portugal) were acquired earlier than rifampicin mutations, although the number of samples harboring these mutations was much smaller. Further studies are necessary to determine whether isoniazid preventive monotherapy, which is one of the treatments for latent tuberculosis, may account for some of this effect, as this could result in a background level of increased isoniazid monoresistance.

Compensatory mutations that potentially ease fitness effects caused by resistance often occur after the evolution of primary resistance. This phenomenon was reviewed by Fonseca et al. [71], and examples include mutations in the *ahpC* promoter region and the *rpoC*/*rpoA* genes, which act as compensatory mutations for isoniazid and rifampicin resistance, respectively. Newer WGS work has pointed to several novel compensatory mutations in *M. tuberculosis*, particularly for rifampicin resistance. Comas et al. [72] identified a set of compensatory mutations in the *rpoB* gene that conferred high competitive fitness in vitro and were also found frequently in clinical populations. In a large-scale analysis of 1000 strains from Russia, Casali et al. [73] examined strains with primary resistance mutations in *rpoB* and identified accompanying compensatory mutations in *rpoA* and *rpoC*. Cohen et al. [69] identified putative rifampicin compensatory mutations that are present in South African strains by searching for *rpoA*, *rpoB*, and *rpoC* mutations that evolved only after or concurrent with rifampicin resistance-conferring mutations. A recent study of highly resistant *M. tuberculosis* strains from Central Asia confirmed that the presence of compensatory mutations, particularly those compensating for the fitness cost of mutations that confer rifampicin resistance, is associated with transmission success and higher drug-resistance rates [74]. Beyond rifampicin resistance compensation, Coll et al. [59] identified mutations in *pncB2* that may compensate for pyrazinamide resistance conferred by *pncA*, and similarly, mutations in *thyX-hsdS.1* (the *thyX* promoter) that may compensate for PAS resistance conferred by *thyA*; however, experimental validation of these potential compensatory relationships is needed. Even fewer studies have identified stepping-stone mutations in *M. tuberculosis*, which emerge prior to higher-level resistance mutations. Cohen et al. [69] found that *ubiA* mutations emerge in a stepping-stone fashion prior to more classic *embB* mutations that confer ethambutol resistance. Safi et al. [15] also showed in vitro that multi-step selection involving *ubiA*, *aftA*, *embB*, and *embC* is required to achieve the highest levels of ethambutol resistance.

### The challenge of mixed infections

Although WGS approaches have great sensitivity in detecting cases of recent transmission, reconstructing the details of transmission networks with WGS [166–168] can be difficult. Transmission network mapping is highly dependent on sampling density and studies rarely, if ever, comprehensively sample an outbreak or the extent of within-host diversity. It is also becoming clear, from the prevalence of very close relationships between isolates from patients who have no other direct epidemiological connections, that transmission may largely result from casual contact in community settings [169]. In addition, the phylogenetic reconstruction of transmission networks can be especially challenging, particularly because of the very close relationships between strains and the slow rate of evolution of *M. tuberculosis* [92, 125–128].

Mixed infections represent a major challenge for understanding drug-resistance evolution within patients [153, 158, 159]. It can be straightforward to disambiguate co-infections of strains from different lineages, but mixed infections involving strains that have few genetic differences can also occur, making these strains difficult to distinguish. Köser et al. [75] used WGS for rapid drug-susceptibility testing of a patient with XDR-TB, and determined that the patient carried two different XDR-TB Beijing strains with differing resistance mutations. In a study by Liu et al. [76], three dominant sub-clones differing by 10–14 SNPs were detected within a single patient, each with different resistance patterns and probably different anatomical distributions. Also, co-infection by strains with differing resistance patterns may yield misleading composite views of resistance; for example, co-infection with two MDR-TB strains—one with quinolone resistance and the other with aminoglycoside resistance—may be mistaken for infection with a single XDR-TB strain.

**Fig. 1:**
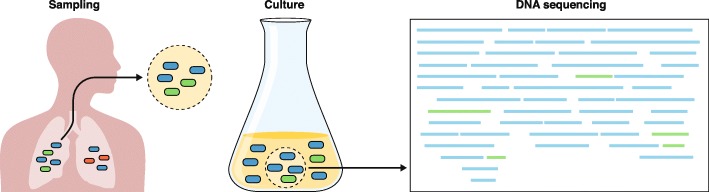
Challenges to predicting drug resistance accurately from clinical specimens using current culture-dependent molecular diagnostics. The left panel depicts an expectorated sputum sample, which may not accurately represent the microbiologic diversity within the source patient. Culturing this sample (center panel) introduces further biases between faster- and slower-growing strains, such that faster-growing strains are over-represented within the cultured sample. Genomic DNA that is isolated and sequenced is input to computer algorithms that determine the genomic content, including the identification of drug-resistance mutations. However, disambiguating samples that contain mixed strains or detecting heteroresistance remains a computational challenge. The left panel was adapted from Ford et al. [170], with permission from Elsevier

Furthermore, newer data suggest that there can be genetic heterogeneity among *M. tuberculosis* isolates from different parts of the body, potentially leading to incomplete views of drug resistance within a patient (Fig. 1). In a study by Lieberman et al. [77], the authors observed evidence for both within-host evolution and mixed infection by piecing together the genetic variation observed among *M. tuberculosis* isolates from multiple post-mortem biopsies from the same patient. Another recent study by Dheda et al. [78] showed that drug concentrations at seven body sites were inversely correlated with the MIC of the bacteria isolated from these sites. Sequencing and comparison to pre-treatment and serial sputum samples suggested ongoing acquired resistance and differential evolution across sites [78]. These findings underscore the limitations of diagnosing or studying the evolution of drug-resistant *M. tuberculosis* using a single patient specimen. However, they also show the promise of WGS for informing interventions related to drug delivery, dosing, and diagnostics, thereby helping to prevent the development of acquired resistance within a patient. More research in this area is needed to determine the breadth and scope of mixed infections among patients with active TB, their contribution to changing drug-resistance patterns over time, and the role of spatial heterogeneity in the evolution of drug resistance.

## From bench to bedside: promise and challenges

Given that the failure to identify and treat patients who have drug-resistant TB leads to increased mortality, spread of resistant strains, and gain of additional drug resistance [171], there is a critical need to diagnose resistant *M. tuberculosis* in patients rapidly. Several important molecular diagnostic platforms have been established for the identification of *M. tuberculosis* and drug resistance within this organism, but they are limited to the identification of a defined subset of resistance mutations [172], do not always include the earliest-arising mutations that precede MDR [66], and do not provide knowledge that is useful in determining whether a patient has been re-infected, whether the patient has a recurrent or mixed infection, or whether a particular infection represents a transmission event. WGS holds significant potential to modernize the TB laboratory and improve upon TB management [173], and this topic has been reviewed previously [173, 174]. To date, WGS has been primarily applied as a clinical tool to achieve two goals: first, to detect *M. tuberculosis* within a clinical sample, and second, to detect resistance mutations and predict resistance patterns so that appropriate treatment can be provided. In order to provide clinically useful information, a diagnostic platform must be rapid. Historically, WGS has relied upon an input of pure mycobacterial cultures, which is time-consuming (requiring multiple weeks) and therefore of less clinical utility. Several investigations have attempted to address this issue by using earlier culture inputs or by attempting culture-independent, direct sequencing from clinical specimens [80, 82, 175]. In a rapid, yet still culture-dependent method, Pankhurst et al. [80] prospectively compared real-time WGS of “early positive liquid cultures” to routine *M. tuberculosis* diagnostics, and found that WGS achieved a faster time to diagnosis at a lower cost.

Although the advances achieved using WGS are promising, several hurdles must be overcome before it can be put into practical use in the clinic (Fig. 1). Requirements for costly equipment, technical expertise, and substantial computational resources present challenges to implementation [173]. Direct sequencing of patient samples has revealed that the vast majority of DNA present is from the patient or from non-mycobacterial prokaryotes, with variable quantities of mycobacterial DNA present. Doughty et al. [81] performed a pilot study demonstrating the feasibility of direct sequencing using a benchtop sequencer (Illumina MiSeq, San Diego, CA) and sputum samples from eight patients. Although they were able to identify the presence of *M. tuberculosis*, the low depth of sequencing coverage of the genome (0.002 to 0.7x) prevented drug-susceptibility prediction. Separately, Brown et al. [176] performed an enrichment step with biotinylated RNA baits prior to direct sequencing of sputum, resulting in higher quality data (> 20x depth and > 90% coverage) that allowed the identification of resistance mutations.

Using a targeted DNA enrichment strategy to study 43 individuals with active pulmonary TB, Doyle et al. [177] compared WGS directly from sputum with mycobacterial growth indicator tube (MGIT) WGS. Although direct sputum sequencing was able to identify drug resistance much more rapidly than MGIT WGS, only 74% of sputum samples yielded interpretable WGS data (vs 100% from MGIT); thus, additional optimization of these methods is needed to increase the sensitivity of this approach. Similarly, in a recent study, the use of pyrosequencing of a concentrated sputum sediment (rather than from sputum directly), dramatically shortened the time to initiation of an MDR-treatment regimen [178].

One promising technology that could change clinical WGS is long-read sequencing using the Oxford Nanopore Technologies (ONT; Oxford, UK) platform. An advantage of ONT is the ability to allow sequencing to continue until sufficient coverage of the genome has been obtained, potentially solving the problem of low or variable amounts of *M. tuberculosis* in clinical samples [82]. Early ONT studies have shown promise in identifying antimicrobial-resistance genes in different bacterial species [179]. Unfortunately, at present, both the high error rate of ONT MinION and potential difficulties with GC-rich regions limit the utility of this technology; thus, improvements in accuracy are necessary to enable the identification of resistance associated with point mutations [179]. ONT metagenomic sequencing has been successfully applied to improve pathogen detection and antimicrobial-resistance testing in other clinical settings [180]; however, to date, applications of this technology to *M. tuberculosis* have been limited to pre-clinical research [82].

Despite these challenges, WGS offers several advantages over the technologies that are currently employed for diagnosis and epidemiological monitoring of TB. Using WGS directly on patient sputum could reduce the turnaround time for diagnosis and determination of antibiotic-resistance status from weeks to hours [61, 159], and would prevent the introduction of culture-induced biases. The depth of information provided by WGS could be used to identify whether an individual harbors multiple co-infecting strains [106, 160] and to distinguish recurrent infection as either relapse or re-infection [174, 181]. In addition, WGS could provide real-time epidemiological information that could be useful for understanding patterns of drug resistance and for establishing chains of transmission [174]. Encouragingly, the high levels of concordance observed between the genotypes and phenotypes of clinical samples indicate that WGS can provide high accuracy for both diagnosing TB and informing treatment options [113]. Finally, WGS of patient samples would provide a high level of convenience, by combining diagnosis, resistance profiling, and epidemiological analysis into a single test [85]. Given these advantages, the WHO has recently published a technical guide for the implementation of next-generation sequencing (NGS) technologies for the detection of drug resistance in *M. tuberculosis* [182].

### Routine whole-genome sequencing of mycobacterial isolates

In 2017, England became the first nation to launch routine WGS of all prospectively identified *M. tuberculosis* clinical isolates [183]. Sponsored by Public Health England (PHE), prospective WGS is being performed on all positive mycobacterial cultures. Within 5–7 days of receipt of the culture from the reference lab, data will be provided on the mycobacterial species, the predicted drug susceptibility, and the molecular epidemiology of the strains. If, from the sequence analysis, a strain is predicted to be fully susceptible to first-line antitubercular drugs, phenotypic drug-susceptibility testing (DST) will no longer be performed routinely. However, if drug resistance to any first-line drug is identified, then phenotypic DST will follow. Beyond drug-susceptibility prediction, these efforts will have profound implications for TB control because WGS data can be used for real-time molecular epidemiology in this context.

Given the high sensitivity of WGS in detecting drug resistance to first-line TB drugs [60], similar algorithms utilizing WGS to predict susceptibility (rather than to identify drug resistance) for first-line drugs, in lieu of phenotypic DST, have been endorsed in the Netherlands and in New York [60]. It seems highly likely that these kinds of efforts would be helpful in higher-burden TB settings than those mentioned here, but the feasibility of this approach has not yet been established, from either a practical or economic standpoint, in settings where the numbers of drug-resistant TB cases are high.

## Conclusions and future directions

Since the first applications of WGS to *M. tuberculosis* in 1998, WGS techniques have greatly accelerated our understanding of drug-resistance mechanisms in this pathogen. Importantly, WGS studies now indicate that, for many drugs, the vast majority of resistance is explained by known mutations. The increasing availability of whole-genome sequences from phenotypically diverse *M. tuberculosis*, combined with improved GWAS algorithms, is enabling the discovery of the remaining determinants of unexplained resistance. In addition, WGS has provided valuable insight into how resistance mutations evolve and spread. It is clear that both de novo acquisition of resistance mutations and clonal transmission are critical factors in the spread of drug-resistant TB.

Furthermore, WGS investigations have revealed that there is a specific order in which drug-resistance mutations are acquired: isoniazid resistance is almost always acquired before rifampicin resistance, which has significant implications for the design of diagnostic tests. Within individual patients, WGS studies have highlighted that mixed infections are common, and often represent important intermediates in the evolution of drug resistance.

WGS also holds great promise for revolutionizing the rapid clinical diagnosis of TB in the future. Although there are still substantial technical hurdles, WGS can be used to diagnose the presence of *M. tuberculosis* rapidly, as well as to pinpoint appropriate antibiotic treatment regimens by identifying the complement of *M. tuberculosis* drug-resistance mutations that are present within a clinical sample. Indeed, improvements in the prediction of drug susceptibility with WGS may obviate the need for phenotypic culture methods, especially for first-line drugs.

Although WGS offers many benefits, targeted NGS, in which sequence data are obtained from only a focused panel of genes or genetic regions rather than from the entire genome, is gaining momentum [184]. One of the advantages of targeted NGS over WGS is that it can be performed directly on clinical specimens and is, therefore, faster than culture-based WGS. Other advantages include reduction in both labor and computational efforts and reduced costs. The potential offered by the application of targeted NGS to the prediction of drug resistance from genomic data is self-evident. Nevertheless, it seems that WGS would have greater discriminatory power than targeted NGS for molecular epidemiology purposes.

Ultimately, the use of WGS is expected to continue to advance our understanding of *M. tuberculosis* drug resistance. Furthermore, its practical use in clinical settings holds great potential to improve public health through real-time molecular epidemiology tracking, to identify global hotspots of drug-resistance emergence, and to facilitate the development of improved approaches for the diagnosis and treatment of drug-resistant TB.

## Abbreviations

DST: Drug-susceptibility testing
GWAS: Genome-wide association study
MDR: Multidrug-resistant
MIC: Minimum inhibitory concentration
NGS: Next generation sequencing
ONT: Oxford Nanopore Technologies
PAS: Para-aminosalicylic acid
RR-TB: Rifampicin-resistant-TB
SNP: Single nucleotide polymorphism
TB: Tuberculosis
WGS: Whole-genome sequencing
WHO: World Health Organization (WHO)
XDR: Extensively drug-resistant
